# Self-Calibrated In-Process Photogrammetry for Large Raw Part Measurement and Alignment before Machining

**DOI:** 10.3390/s17092066

**Published:** 2017-09-09

**Authors:** Alberto Mendikute, José A. Yagüe-Fabra, Mikel Zatarain, Álvaro Bertelsen, Ibai Leizea

**Affiliations:** 1 IK4-Ideko, 20870 Basque Country, Spain; mmzatarain@gmail.com (M.Z.); abertelsen@gmail.com (Á.B.); ileizea@ideko.es (I.L.); 2 I3A, Universidad de Zaragoza, 50018 Zaragoza, Spain; jyague@unizar.es

**Keywords:** image, machine tool, photogrammetry, calibration

## Abstract

Photogrammetry methods are being used more and more as a 3D technique for large scale metrology applications in industry. Optical targets are placed on an object and images are taken around it, where measuring traceability is provided by precise off-process pre-calibrated digital cameras and scale bars. According to the 2D target image coordinates, target 3D coordinates and camera views are jointly computed. One of the applications of photogrammetry is the measurement of raw part surfaces prior to its machining. For this application, post-process bundle adjustment has usually been adopted for computing the 3D scene. With that approach, a high computation time is observed, leading in practice to time consuming and user dependent iterative review and re-processing procedures until an adequate set of images is taken, limiting its potential for fast, easy-to-use, and precise measurements. In this paper, a new efficient procedure is presented for solving the bundle adjustment problem in portable photogrammetry. In-process bundle computing capability is demonstrated on a consumer grade desktop PC, enabling quasi real time 2D image and 3D scene computing. Additionally, a method for the self-calibration of camera and lens distortion has been integrated into the in-process approach due to its potential for highest precision when using low cost non-specialized digital cameras. Measurement traceability is set only by scale bars available in the measuring scene, avoiding the uncertainty contribution of off-process camera calibration procedures or the use of special purpose calibration artifacts. The developed self-calibrated in-process photogrammetry has been evaluated both in a pilot case scenario and in industrial scenarios for raw part measurement, showing a total in-process computing time typically below 1 s per image up to a maximum of 2 s during the last stages of the computed industrial scenes, along with a relative precision of 1/10,000 (e.g., 0.1 mm error in 1 m) with an error RMS below 0.2 pixels at image plane, ranging at the same performance reported for portable photogrammetry with precise off-process pre-calibrated cameras.

## 1. Introduction

Large parts machining is performed on a near-to-shape raw part, obtained by processes like casting or welding. These raw parts very often do not have any reliable surface or feature reference that can be used for in-machine alignment. However, initial alignment of the part at the machine is a critical process, since an incorrect alignment will give rise to material shortage, which is associated with spoiling the part or with a costly recovery process. Due to the high cost associated with the rejection of a part, the initial alignment is usually done by way of long, time consuming manual processes.

**Figure 1:**
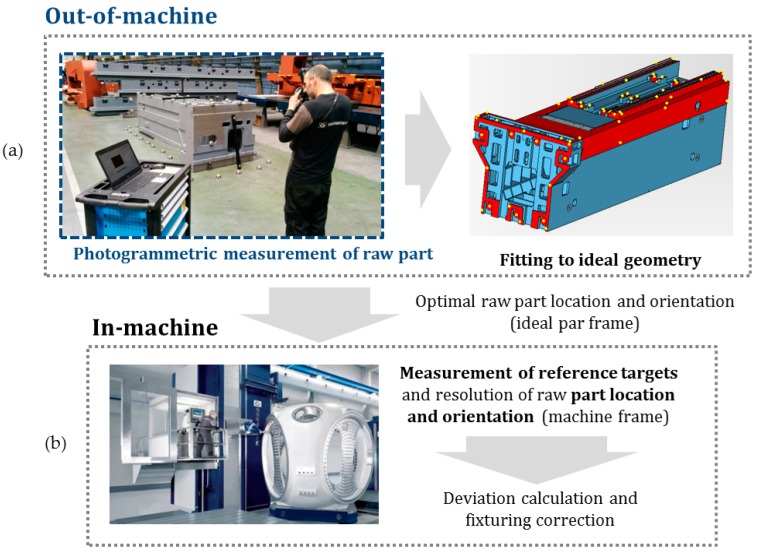
Portable photogrammetry for out-of-machine raw part measurement (**a**) for efficient in-machine alignment process (**b**) of large components prior to machining.

Photogrammetry methods are being used more and more as a 3D technique for large scale applications [1,2,3,4]. To overcome the aforementioned limitations of the large raw part manual alignment, a photogrammetry based process was developed in previous work [5]. The process consists of two steps (Figure 1): (a) Out-of-machine measurement by photogrammetry of optical targets on raw part surfaces, and its mathematical orientation (fitting) to the ideal designed part frame, where the optimal location of optical targets is obtained to use them as feature references in the machine, and (b) In-machine measurement of such references by a machine-integrated measurement system, a special purpose machine vision or a spindle-integrated contact probe, where the in-machine location and orientation of the raw part is measured, assisting the machine operator through an efficient alignment and part fixturing process prior to part machining.

Two main limitations were observed for the out-of-machine portable photogrammetry presented in [5]: First, since a post-process ray-net bundle adjustment [6] was adopted, long computing times were needed for solving the 3D target coordinates after taking an image set, being in the range of some minutes. Due to the post-process computing approach, the lack of adequate images for having a solvable and consistent multiple view geometry or the diagnosis of pending optical targets to measure, could only be performed after all images were taken [7,8]. This resulted in a time consuming and user dependent iterative review and re-process measuring procedure until an adequate set of images was completed. As a result, the measuring process was clearly far away from the potential that photogrammetry may bring to easy, low-cost and fast industrial metrology for large components [9]. Better in-process computational efficiency was required for solving the bundle adjustment, so that images taken together thus far with solved 3D target coordinates could be checked-out in quasi real time for a reliable measuring process, making the most of the potential of portable photogrammetry in large scale applications.

Second, the system was thoroughly tested in a pilot case scenario showing measuring errors in the range of 1 mm for 1 m long parts (1/1000 relative precision), mainly due to the lack of precise calibration of the camera. Better accuracy was necessary for gaining performance when measuring raw parts larger than 1 m where the errors would increase proportionally to the size, as well as for applications where tighter raw material clearances may be expected. Indeed, camera calibration is a relevant uncertainty contributor in photogrammetry [2,10,11], and precise measurements require precise camera model calibration (so called intrinsic parameters). Continuous and recent improvements regarding the mathematical analysis of the data taken for calibration [11,12,13] and image distortion correction [14,15] can be found in the literature.

In the present work, a new efficient procedure is presented for solving the bundle adjustment problem in portable photogrammetry. The presented development demonstrates efficient in-process bundle computing capability on a consumer grade desktop PC, enabling quasi real time 2D image and 3D scene diagnosis so that a reliable measuring procedure can be conducted by portable photogrammetry, avoiding user dependent and inefficient measuring procedures. Additionally, based on the efficient computational approach, an in-process self-calibration method is implemented using the solved 3D point cloud scene itself as calibration geometry, avoiding the need for specialized off-process pre-calibrated cameras or the use of special purpose calibration artifacts in the scene for precise measurements in an industrial metrology application, where traceability is set only by the scale bars available in the measuring scene.

The developed self-calibrated in-process photogrammetry has been evaluated both in the pilot case scenario of [5] (1.5 m long reference part) and at two large scale industrial scenarios (up to 15 m long raw parts), showing successful results with an in-process computing time ranging around 1 s per image and a relative precision of 1/10,000, which is at the precision level reported for portable photogrammetry with precise pre-calibrated cameras.

Section 2 below describes the raw part measuring process by photogrammetry, along with the adopted method for evaluating measurement accuracy both in the pilot case and industrial scenarios. Section 3 introduces the multiple view geometry in portable photogrammetry, along with the non-linear multivariable optimization problem to compute the bundle adjustment. Section 4 presents the implemented in-process image and target computing procedure, and Section 5 describes the self-calibration functionality development, where computing efficiency and precision performance are demonstrated for the pilot case under study, respectively. Section 6 shows the evaluation results for large scale industrial scenarios. Finally, main conclusions and further steps are described in Section 7.

## 2. Materials and Methods

Regarding precision in portable photogrammetry, the uncertainty determination of optical measurement methods is still an issue [16]. Analytical calculated uncertainty budget of photogrammetric systems according to GUM [17] using error propagation theory is a challenge and consistent approaches have not been found in the literature. The main reason is the large number of undefined error sources affecting the process, especially when not working in laboratory conditions [1,2]. Variability simulation is an alternative to uncertainty budgeting for complex measurement tasks. However, it also requires deep knowledge of the measurement chain and the statistical distribution of each influence quantity [18], which is also a challenging issue in photogrammetry [19]. Therefore, despite the existence of well-accepted guidelines and standards, the definition of accuracy performance is still very heterogeneous [20]. However, industrially accepted assessment guidelines such as the VDI 2634 [21] offer an alternative for evaluating photogrammetric measurement performance, where the recommended procedure is to arrange a set of calibrated scale bars (control bars) around the object scene and through the principal coordinate axes. Lengths measured by photogrammetry are compared to their calibrated values and corresponding length measuring errors (LME) are then evaluated. 

**Figure 2:**
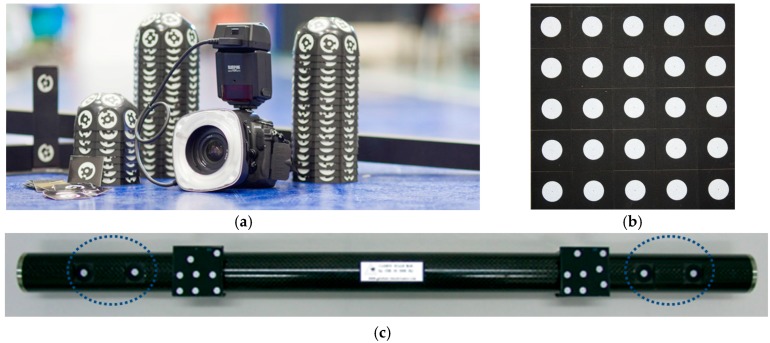
Measuring instrumental in portable photogrammetry (**a**), including a fixed focal consumer grade digital camera (Nikon D300S, 4288 × 2848 pixels, 24 mm), measuring frame, and multicoded target artifacts for efficient image matching assistance; (**b**) non-coded targets (Geodesie, diameter 8 mm) [22] for raw part measurement; and (**c**) carbon-fiber scale (Geodesie) [22] for precise traceability and precision evaluation, with precalibrated distances between non-coded targets (blue).

Photogrammetry is the core technique used in the procedure presented in [5] for raw part alignment into a machine tool prior to its machining (Figure 1). The raw part is measured by photogrammetry, using retroreflective coded and non-coded optical targets. Non-coded targets (Figure 2) are used to measure the raw part surfaces to be machined. Auxiliary coded targets are used to assist the measuring process by portable photogrammetry so that consistent image-taking is performed during measurement, enabling the correspondence matching between the information given at consecutive images. Images are taken around the part by a digital camera, and transferred in-process to a desktop PC via wireless communication. Images are computed and optical target 3D coordinates are obtained. After measuring, non-coded target coordinates are fitted to the desired nominal part geometry (defined in a CAD file in this work), using a specific purpose computing criteria for fitting [5], enabling even and positive overstock distribution over the surfaces to be machined. As a result, available overstock over all raw part surfaces can be controlled prior to part set-up into the machine. After out-of-machine measurement and fitting finishes, a subset among the optical target 3D coordinates are selected and used as a reference for raw part in-machine alignment. An in-machine measuring system is then used for measuring the selected target coordinates in the machine frame, and the raw part fixturing is adjusted so that its alignment to the machine axes corresponds with its computed optimal by fitting.

**Figure 3:**
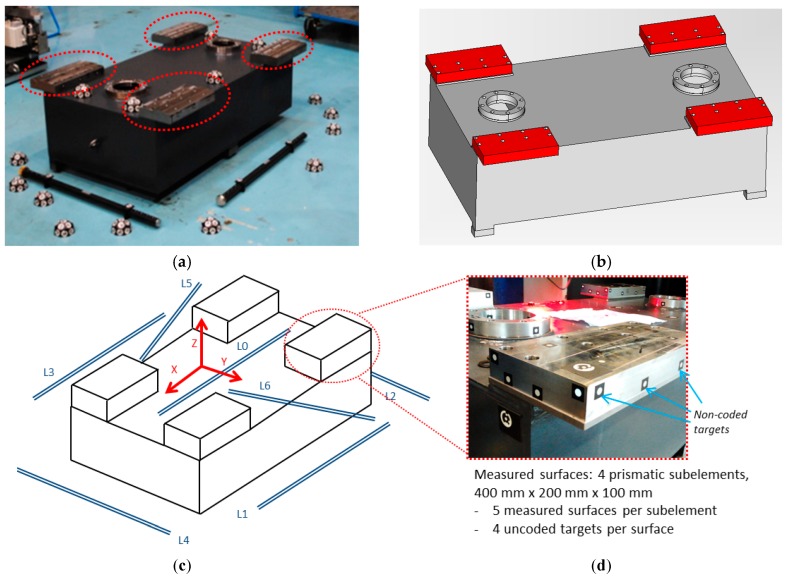
Steel raw part pilot case (**a**) under analysis (1.5 m × 1 m × 0.5 m) with prismatic subelements (in red, 400 mm × 200 mm × 100 mm) milled to a 0.01 mm precision, with multicoded targets around the scene for assisting image matching; (**b**) CAD describing nominal reference surfaces (in red), and (**c**) uncertainty evaluation scene and scale bar lay-out for LME evaluation, with non-coded targets on surfaces to measure (**d**).

Figure 3 shows the verification scenario adopted in this work in order to evaluate the precision of the developed self-calibrated portable photogrammetry. A reference part is adopted as a pilot case (Figure 3a), resembling the measuring scenario at [5] where limited relative precisions of 1/1000 were reported for portable photogrammetry, with four prismatic steel subelements screwed to a mechano-welded structure and milled to a nominal geometry (Figure 3b). A length measuring error (LME) approach similar to VDI 2634 has been adopted for evaluating measuring procedure uncertainty in the developed self-calibrated portable photogrammetry, verifying the performance of the corresponding measurands (i.e., optical target 3D coordinates). Six scale bars (L1 to L6) were set around the scene (1.5 m × 1 m × 0.5 m) in two groups of three (L1 to L3, and L4 to L6), along with a scale bar in the center of the scene (L0), up to a total of seven bars in order to analyze uncertainty in non-coded target measuring (Figure 3c). Each group of three was located in opposite corners of the scene and each scale bar in a group was aligned such that the length components were set in all three spatial directions. For avoiding the influence of length calibration uncertainty between different scale bars in the LME analysis, only one and same scale bar, a calibrated carbon fiber bar with two non-coded targets separated a length L_scale_ = 1340.099 mm, was used and moved around the scene to each location. Additionally, as an alternative way of evaluating measuring performance, prismatic subelements (Figure 3d) have been measured placing non-coded targets on each milled surface. 3D coordinates of the computed non-coded targets were fitted to its nominal geometry, and the errors corresponding to each nominal reference surface were evaluated.

Eventually, the self-calibrated in-process photogrammetry was evaluated in two industrial scenarios with up to 15 m long raw parts, having as a reference a spindle integrated contact probe measuring process (repeatability ranging 1 μm) executed in relatively accurate milling machines (assumed uncertainty ranging at a typical value of 0.01 mm). Raw part surfaces to be machined were measured and non-coded target coordinates fitted [5] to its nominal. A subset among the fitted target coordinates (control points) were then used as a reference for raw part in-machine alignment, measured by a machine-spindle integrated contact probe during the alignment process. Once the part was properly aligned, machine coordinates of optical targets not used as control points for alignment (i.e., check points) were measured by the contact probe. In order to evaluate the measuring performance of the developed self-calibrated photogrammetry, errors were evaluated between the gauged values by the contact probe and the corresponding 3D coordinates obtained after measurement and fitting.

## 3. Measurement by Portable Photogrammetry

Photogrammetry has been defined by the American Society for Photogrammetry and Remote Sensing (ASPRS) [23] as the art, science, and technology of obtaining reliable information about physical objects and the environment through processes of recoding, measuring and interpreting photographic images and patterns of recorded radiant electromagnetic energy and other phenomena. In precise industrial metrology applications, it calculates the 3D coordinates of a discrete number of optical targets on an object [20,24] based on their 2D image coordinates. Several photographs of an object are obtained from different positions of the camera. By detection of optical target 2D coordinates at different images it is possible to calculate the positions and orientations of the camera for each of the photographs (so called extrinsic parameters), and also to calculate the 3D coordinates of the target points by multiple view triangulation. The measuring frame is given by a predefined set of coded optical targets with initially known 3D coordinates (Figure 2) and process traceability is set by appropriate scale bars (Figure 2) with calibrated lengths between corresponding pairs of optical targets.

### 3.1. Multiple-View Geometry

**Figure 4:**
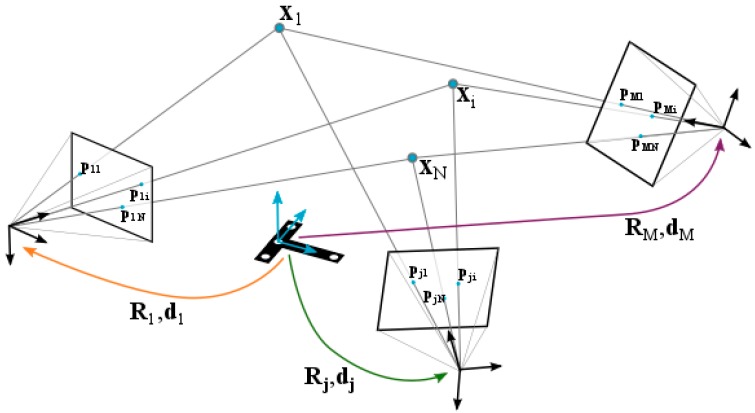
Epipolar ray-net geometry in portable photogrammetry. Camera frame coordinates (*R_j_* and *d_j_*) and optical target 3D coordinates ($X_{i}$) can be expressed with respect to a predefined measuring frame, and computed according to the ray-net corresponding to a set of projected 2D target coordinates (*p_ij_*) at each image plane.

The geometry of a general photogrammetric problem is illustrated in Figure 4. A set of *i =* 1 … *N* targets with 3D coordinates $X_{i}$ are projected in the image plane *p_ij_* from *j =* 1 … *M* different points of view. Each camera position has associated extrinsic parameters, given by the rotation matrix *R_j_* defined by the rotations around the reference frame axes *x*, *y*, *z*-based on the *α_j_*, *β_j_* and *γ_j_* Euler angles- and the translation vector *d_j_*. The main goal of portable photogrammetry is the joint determination of the 3D coordinates of the *N* targets ($X_{i}$) along with the location and orientation (camera extrinsic parameters, *R_j_* and *d_j_*) of the *M* image camera frames. 

Each target 3D coordinates $X_{i}={\left[x_{i}\;y_{i}\;z_{i}\right]}^{T}$ can be expressed as $U_{ij}={\left[u_{ij}\;v_{ij}\;w_{ij}\right]}^{T}$ in each camera frame depending on its extrinsic parameters *R_j_* and *d_j_* as:

(1) $$U_{ij}=R_{j}X_{i}+d_{j}$$

**Figure 5:**
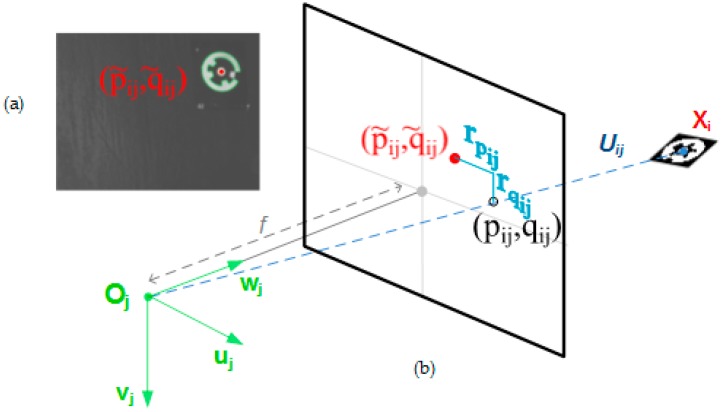
Target 2D detection and conic projection through epipolar line. (**a**) Example of optical target coordinate detection (${\tilde{p}}_{ij}$ and ${\tilde{q}}_{ij}$) at image plane; (**b**) Conic projection ($p_{ij}$ and $q_{ij}$) into image plane of the target 3D coordinate $X_{i}$ according to epipolar line from $O_{j}$ camera frame (*R_j_* and *d_j_*), with the corresponding projection error contribution ($r_{p_{ij}}$ and $r_{q_{ij}}$) to the joint residual error.

Each target 3D coordinate $U_{ij}$ can be projected into the corresponding camera 2D image plane as $p_{ij}$ and $q_{ij}$ coordinates (Figure 5), following the widely assumed pin-hole conic projection model in machine vision [24] as:

(2) [pijqij]=f[uijwijvijwij]

being *f* the focal distance of the camera lens. 

The widely assumed Brown’s model [1,16,25] is adopted for modelling camera and lens distortions, which can be expressed as follows:

(3) $$\left[\begin{array}{c} \tilde{p_{ij}}\\ \tilde{q_{ij}} \end{array}\right]=\left[\begin{array}{c} h_{ij}\left(1+k_{1}r_{ij}^{2}+k_{2}r_{ij}^{4}\right)+\pi_{1}\left(r_{ij}^{2}+2h_{ij}^{2}\right)+2\pi_{2}h_{ij}v_{ij}\\ v_{ij}\left(1+k_{1}r_{ij}^{2}+k_{2}r_{ij}^{4}\right)+\pi_{2}\left(r_{ij}^{2}+2v_{ij}^{2}\right)+2\pi_{1}h_{ij}v_{ij} \end{array}\right]\;r_{ij}=\sqrt{{\left(h_{ij}-h_{0}\right)}^{2}+{\left(v_{ij}-v_{0}\right)}^{2}}$$

where ($h_{ij},\;v_{ij}$) are the 2D detected target coordinates in the image plane and $\left(\begin{array}{c} \tilde{p_{ij},\;} \end{array}\tilde{q_{ij}\;}\right)$ are the undistorted or corrected image coordinates, given $k_{1}$ and $k_{2}$ as the first and second order coefficient for modelling the radial distortion, ($h_{0}$,$v_{0}$) as the center of distortion and $\pi_{1}$ and $\pi_{2}$ as the first order tangential distortion coefficients. 

The camera and lens distortion model, along with the focal distance, form the so called camera intrinsic parameters characterizing the camera condition for multiple view geometry. The camera intrinsic parameters can be obtained by previous off-line calibration processes [26,27]. 

Reprojection residual errors $r_{p_{ij}}=\left(p_{ij}-\tilde{p_{ij}}\right)$ and $r_{q_{ij}}=\left(q_{ij}-\tilde{q_{ij}}\right)$ can then be defined for every target observed at every image (Figure 5) as the difference between the corrected target coordinates ${\tilde{p}}_{ij}$ and ${\tilde{q}}_{ij}$ and the projected target coordinates $p_{ij}$ and $q_{ij}$, which directly depend on the 3D coordinates of the targets ($X_{i}$) and camera extrinsic parameters (*R_j_* and *d_j_*) to be solved. As a result, given by the camera intrinsic parameters and the multiple view geometry, target 3D coordinates and camera extrinsic parameters can be jointly computed so that residual errors are minimized for every target at every image, leading to the so-called bundle adjustment [24] for solving the reprojection optimization problem in portable photogrammetry.

### 3.2. Optimization Problem

The problem is thus defined as a nonlinear multivariate overdetermined system, which can be solved as a least square optimization problem or bundle adjustment by numerical methods such as Levenberg-Marquardt or Gauss-Newton [6,20,24,25]. An initial approximation is defined for the set of variables to solve (*α_j_*, *β_j_*, *γ_j_*, and *d_j_* from *j =* 1 … *M* camera views, and $X_{i}$ from *i =* 1 … *N* optical targets) and a numerical iteration procedure is applied to compute them so that residual errors ($r_{p_{ij}}$ and $r_{q_{ij}}$) are minimized.

Residual errors can be grouped into a single residual vector *r* according to the following structure:

(4) $$r_{j}=\left[\begin{array}{c} r_{11}\\ r_{11}\\ \vdots \\ r_{1Nj}\\ r_{2Nj} \end{array}\right]=\left[\begin{array}{c} p_{1}-\tilde{p_{1}}\\ q_{1}-\tilde{q_{1}}\\ \vdots \\ p_{Nj}-\tilde{p_{Nj}}\\ q_{Nj}-\tilde{q_{Nj}} \end{array}\right]\;r=\left[\begin{array}{c} r_{1}\\ \vdots \\ r_{M} \end{array}\right]$$

where each *r_j_* vector corresponds to the reprojection error distribution at the *j* image, being *N_j_* the number of targets detected in each image.

There are two main sets of parameters to be solved-extrinsic and target coordinates, represented by *θ_e_* and *θ_x_*, respectively, which can be grouped in a common vector *θ*.

The extrinsic parameters vector *θ_e_* is represented as follows,

(5) $$\theta_{e}={\left[\alpha_{1}\;\;\beta_{1}\;\gamma_{1}\;d_{1}^{T}\;\ldots \;\alpha_{M}\;\;\beta_{M}\;\gamma_{M}\;d_{M}^{T}\right]}^{T}$$

and the target coordinates vector *θ_x_* is like

(6) $$\theta_{x}={\left[x_{1}\;\;y_{1}\;z_{1}\;\ldots \;x_{N}\;\;y_{N}\;z_{N}\;\right]}^{T}$$

Based on these definitions, the complete vector *θ* is defined as

(7) $$\theta =\left[\begin{array}{c} \theta_{e}\\ \theta_{x} \end{array}\right]$$

Following the above definitions, the process consists in the iteration of vector θ ($\theta_{k}\leftarrow \theta_{k-1}+{∆}_{\theta }$) towards an optimal value $\hat{\theta }$ which minimizes the residual vector ${\left\|\vec{r}\right\|}^{2}$ norm. According to Gauss-Newton method [28], each iteration ${∆}_{\theta }$ can be computed as a linear system by

(8) $$J^{T}J{∆}_{\theta }=-J^{T}r$$

where *J* is the Jacobian matrix containing the partial derivatives of each component of the residual vector with respect to the parameters to optimize in θ.

(9) $$J\left(\theta \right)=\left[\begin{array}{ccc} \frac{\partial r_{1}}{\partial \theta_{1}} & \ldots & \frac{\partial r_{1}}{\partial \theta_{6M+3N}}\\ \vdots & \ldots & \vdots \\ \frac{\partial r_{2m}}{\partial \theta_{1}} & \ldots & \frac{\partial r_{2N}}{\partial \theta_{6M+3N}} \end{array}\right]$$

with $m=\overset{M}{\underset{j=1}{\sum }}N_{j}$ being the total number of targets detected at all images.

## 4. In-Process Computing Procedure for Time Efficiency

The computing performance of the bundle adjustment method plays a relevant role in the development of a time-efficient in-process strategy for portable photogrammetry. The convergence time of the numerical method depends on the size of the Jabobian matrix J, due to matrix element allocation, assignation and computation times for managing relatively large and sparse $J^{T}J$ and $J^{T}$ matrices, and due to the conditioning and size of the ${\left(J^{T}J\right)}^{-1}$ inverse matrix calculation so that ${∆}_{\theta }$ is determined in each iteration. As an example, given a measuring scenario with 100 images and 100 targets, assuming all targets are detected at every image, $J^{T}J$ matrix size is 900 × 900, with J being 40,000 × 900. Indeed, this problem increases with the number of images and targets included in the joint bundle during the measuring process. An alternative for reducing this computational work is the decomposition of the linear system to solve ${∆}_{\theta }$ (Equation (8)) to a set of a lower range and individually solved linear subsystems. In this work, the reprojection error partial derivatives forming the Jacobian matrix J (Equation (9)) are analytically expressed and system decomposition is adopted so that ${∆}_{\theta_{e}}$ and ${∆}_{\theta_{x}}$ are individually solved for interdependent extrinsic and target coordinate iteration, avoiding direct computation of ${∆}_{\theta }$ in order to increase computational efficiency.

On the other hand, the total number of iterations (*k*) and the corresponding convergence time depends on the initial adopted approximation for every variable in θ to optimize in the joint bundle minimization problem. Furthermore, the numerical method may diverge if a too-inaccurate initial approximation is adopted. So, along with the former system decomposition approach, the adoption of adequate and robust initial approaches for new camera extrinsic and target coordinates every time a new image is taken can avoid unnecessary joint computation effort every time a new image is taken.

**Figure 6:**
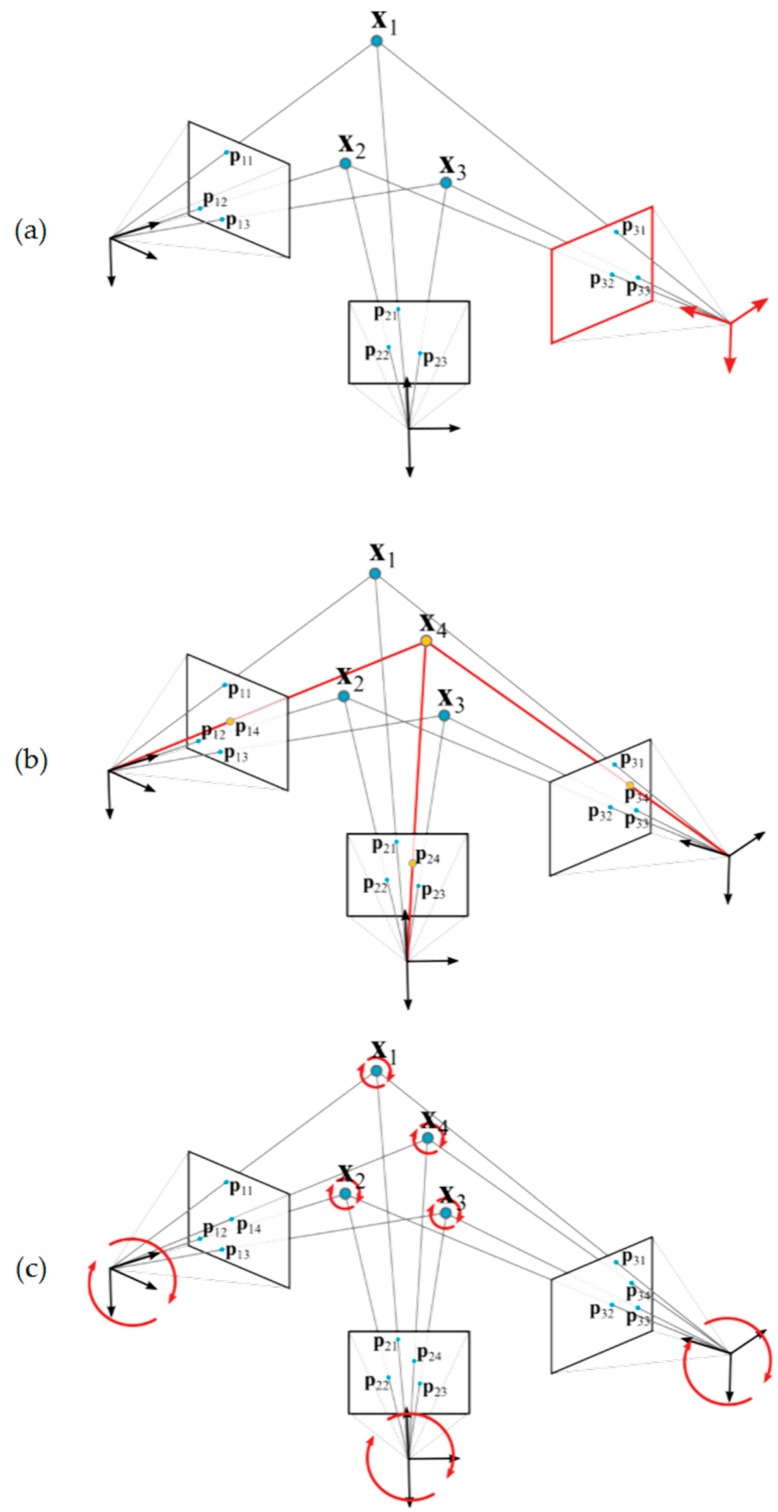
In-process computing procedure. Once a new image is processed, the extrinsic parameters are solved according to X_1_, X_2_, and X_3_ target coordinates (**a**). Then, the 3D coordinates of a new target (X_4_) is solved given by different points of view with known camera extrinsic parameters (**b**); Finally, camera extrinsic parameters and target coordinates are jointly optimized in an in-process bundle adjustment (**c**).

Figure 6 shows a schematic view of the in-process procedure developed. The following steps are conducted each time a new image is taken during the measuring process by portable photogrammetry:

1. Image processing proceeds for optical target detection (*h_ij_*, *v_ij_*) and correction $(\tilde{p_{ij}},\tilde{\;q_{ij}}$), along with decoding of coded targets [24,29].
2. Computation of an initial approach is performed for the camera extrinsic parameters of the new image (*α_j_*, *β_j_*, *γ_j_*, and *d_j_*), according to already solved coded optical target 3D coordinates detected on the image (Figure 6a).
3. In case a minimum set of three coded optical targets with known coordinates is not available in the new image, the camera extrinsic is not computed and the procedure stops asking for a new image having a minimum set of targets to proceed back in step 1.
4. Given by the new camera extrinsic, code assignation is performed to non-coded targets with still unsolved correspondences in all images captured so far, following the so called Hungarian method [30,31].
5. Given by the new camera extrinsic parameters, computation of an initial approach is performed for new target 3D coordinates $X_{i}$ unsolved so far but coded, provided that each one is jointly observed by a minimum set of two camera views with known extrinsic parameters (Figure 6b).
6. An intermediate joint bundle adjustment (Figure 6c) is conducted for the camera extrinsic and target coordinates solved according to all images so far. Given by the initial approaches in steps 2 and 5, only one bundle iteration is performed, so that a sufficiently accurate and consistent epipolar net construction is obtained every time a new image is included in the minimization problem, ensuring a reliable correspondence solving for non-coded targets in step 4, and avoiding unnecessary computational work until joint bundle convergence at this step. The measuring process can now continue with the acquisition of new images, computed in-process from step 1 to 6 every time a new image is taken.
7. Finally, once the measuring process finishes, the post-process joint bundle of camera extrinsic parameters and target coordinates is computed until convergence and measuring process traceability is set by calibrated scale bar distances available at the scene, where measuring frame target coordinates are also included into the bundle adjustment.

Regarding process reliability, step 3 enables in-process control of the information provided by each image, so that measuring process by portable photogrammetry can be guided ensuring a set of images that will form a consistent and geometrically determined ray-net, avoiding inefficient post-process evaluation and iterative processes [7,8]. 

The main steps of the presented in-process strategy are described below. Results are shown for the pilot case under study (Figure 3). Section 4.1 and Section 4.2 show the initial computation approach of the camera’s extrinsic parameters (step 2, Figure 6a) and target coordinates (step 5, Figure 6b) as independent computing problems, respectively. Section 4.3 and Section 4.4 describe the joint bundle adjustment problem both before (step 6, Figure 6c) and after, including scale bars for traceability (step 7), respectively. Special attention is paid to the particular analytic expression of Equation (9) corresponding to each step of the in-process procedure. Although linear closed form expressions can be given in Section 4.1 and Section 4.2 for the independent camera extrinsic parameters and target coordinate computing [24], nonlinear approaches are shown as intermediate steps towards presenting the submatrix decomposition of Equation (8) for the nonlinear joint bundle computation described in Section 4.3 and the following. Optical target image coordinate detection ($h_{ij}$,$v_{ij}$) and decoding of coded targets is performed following [29].

### 4.1. Camera Extrinsic Parameters Initial Approach Computation

Computation of the extrinsic parameters of a *j*-th camera view (Figure 6a) means determining vector $\theta_{e_{j}}$, where camera principal frame location ($d_{x}$, $d_{y}$, and $d_{z}$) and orientation (α, β, and γ Euler angles defining a corresponding rotation matrix $R_{j}$ as $R_{\gamma }R_{\beta }R_{\alpha }$) are defined with respect to a measuring frame (Figure 4) determined by a pre-set of optical coded targets. The detection of a minimum set of 3 targets is required in order to geometrically determine image extrinsic computing.

(10) $$\theta_{e_{j}}={\left[\alpha \;\beta \;\gamma \;d_{x}\;d_{y}\;d_{z}\right]}^{T}$$

As inputs for the method, *X_i_* coordinates of the set of coded reference targets are known, as well as camera intrinsic parameters (focal distance, and distortion model parameters for Equation (3)). As a result, partial derivatives (Equation (9)) in a Jacobian matrix $E_{j}$ can be defined as follows (Equation (11)) for solving camera extrinsic parameters for the *j*-th image as a nonlinear reprojection minimization problem.

(11) $${\left(E_{j}\right)}_{2N_{j}\times 6}=\left[\begin{array}{c} J_{e_{1j}}\\ \vdots \\ J_{e_{Nj}} \end{array}\right]$$

where each submatrix $J_{e_{ij}}$ contains the partial derivatives of the projection errors $r_{p_{ij}}=\left(p_{ij}-\tilde{p_{ij}}\right)$ and $r_{q_{ij}}=\left(q_{ij}-\tilde{q_{ij}}\right)$ of the *i*-th target *X_i_* coordinates with respect to the *j*-th image camera extrinsic, to a total of *N_j_* optical targets detected in the that *j*-th image. Each $J_{e_{ij}}$ submatrix can be expressed as

(12) $${\left(J_{e_{ij}}\right)}_{2\times 6}={\left(E_{ij}\right)}_{2\times 6}=D_{P}D_{U_{E}}$$

where *D_P_* contains the partial derivatives of the *i*-th projected target coordinates $p_{ij}$ and $q_{ij}$ in the *j*-th image with respect to $U_{ij}$ target coordinates (Equation (1)) in the corresponding *j*-th camera principal frame, given as follows according to Equation (2)

(13) $$D_{P}=\left[\begin{array}{ccc} \frac{1}{w_{ij}} & 0 & -\frac{u_{ij}}{w_{ij}^{2}}\\ 0 & \frac{1}{w_{ij}} & -\frac{v_{i}}{w_{ij}^{2}} \end{array}\right]$$

and where $D_{U_{E}}$ expresses the partial derivatives of the $U_{ij}$ target coordinates with respect to the *j*-th camera extrinsic in $\theta_{e_{j}}$ as

(14) $$D_{U_{E}}=\left[\;D_{A}X_{i}\;D_{B}X_{i}\;D_{C}X_{i}\;I_{3\times 3}\right]\;D_{A}=R_{\gamma }R_{\beta }D_{\alpha }\;D_{B}=R_{\gamma }R_{\beta }R_{\alpha }\;D_{C}=D_{\gamma }R_{\beta }R_{\alpha }$$

given the partial derivatives $D_{A}$, $D_{B}$, and $D_{C}$ of the camera principal frame rotation matrix *R* with respect to each Euler angle α, β, and γ, respectively.

**Figure 7:**
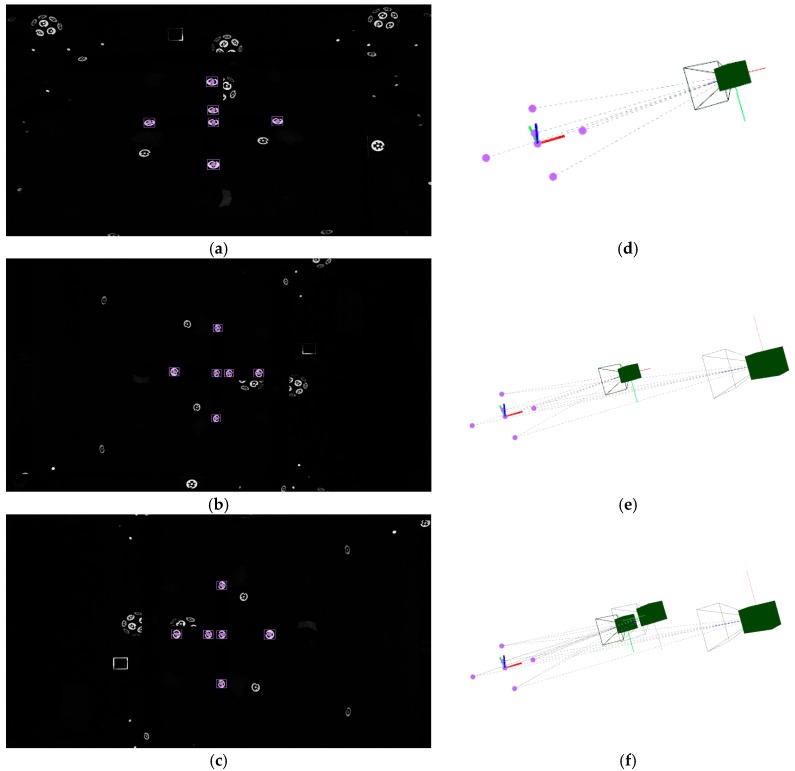
Computing of initial approach of a new camera extrinsic parameters. First set of three images is shown (**a**–**c**), where coded targets on measuring reference frame are detected (highlighted in purple on each image). Camera frames according to computed extrinsic parameters are depicted in (**d**,**e**), and (**f**) corresponding to each image (**a**–**c**), respectively, along with the epipolar net for each image to the set of detected targets in the measuring reference frame (purple).

At the beginning of the measuring process by portable photogrammetry, first images are taken to the set of coded targets determining the measuring frame. Given that a minimum number of three targets are detected in an image, the corresponding individual iteration of $∆\theta_{e_{j}}$ and independent extrinsic computing can proceed for each image. Figure 7 shows an example of computation of the extrinsic initial approach for the first three images of a measuring process conducted on the pilot case evaluation scenario (Figure 3). Nominal values are adopted as an initial approach for the intrinsic parameters, with focal distance ***f*** being 24.0 mm and first order radial distortion coefficient $k_{1}$ being 5.0 × 10^−9^ pixel^−2^, where higher order radial distortion coefficients are neglected, along with decentering and tangential distortion coefficients in Equation (3). At this stage, the initial adopted approach for the intrinsic parameters is not intended to be accurate but only a nominal value close enough to the expected precise one, to be computed by the in-process self-calibration (see Section 5) so that the final bundle converges in a stable and efficient way. 

Table 1 presents the known 3D coordinates (*X_i_*, *i* = 1 ..6) of the detected coded targets on each image, along with the detected image coordinates for each target at each image ($h_{ij}$,$v_{ij}$, *j =* 1 .. 3).

Table 2 shows the computed extrinsic parameters for each camera view, according to Gauss-Newton method following Equation (8) for $\theta_{e_{j}}$ iteration. The extrinsic computing performance can be observed in the resulting RMS value of the reprojection error vector after convergence, ranging at 0.5 pixels. Regarding computing efficiency, a mean time lower than 0.1 ms is observed for each iteration.

### 4.2. Target 3D Coordinate Initial Approach Computation

Once the camera extrinsic parameters are available (Table 2), 3D coordinates of new targets other than those defining the measuring frame can be computed. Analog to extrinsic computing in Section 4.1, the computation of the 3D coordinates of a *i*-th new target (Figure 6b) means determining vector $\theta_{x_{i}}$, where *X_i_* coordinates are defined in the same measuring frame at which camera extrinsic parameters are known. The detection of the target in a minimum set of 2 images is required in order to geometrically determine target coordinate computing by triangulation.

(15) $$\theta_{x_{i}}=X_{i}={\left[x\;y\;z\right]}^{T}$$

As inputs for the method, camera intrinsic and extrinsic parameters (*α_j_*, *β_j_*, *γ_j_*, and *d_j_*) of a minimum set of images are known. As a result, partial derivatives (Equation (9)) in a Jacobian matrix $DX_{i}$ can be defined as follows (Equation (16)) for solving 3D coordinates for the *i*-th target.

(16) $${\left(DX_{i}\right)}_{2M_{i}\times 3}=\left[\begin{array}{c} J_{x_{1i}}\\ \vdots \\ J_{x_{Mi}} \end{array}\right]$$

where each submatrix $J_{x_{ij}}$ contains the partial derivates of the projection errors $r_{p_{ij}}$ and $r_{q_{ij}}$ of the *i*-th target with respect to its *X_i_* coordinates, to a total of *M_i_* images in which the *i*-th target is detected. Each $J_{x_{ij}}$ submatrix can be expressed as

(17) $$J_{x_{ij}}={\left(X_{ij}\right)}_{2\times 3}=D_{P}D_{U_{X}}$$

where *D_P_* was defined in Equation (13), and $D_{U_{X}}$ expresses the partial derivatives of the $U_{ij}$ target coordinates at the *j*-th camera frame with respect to its $X_{i}$ coordinates at the common measuring frame as

(18) $$D_{U_{X}}=R_{j}$$

being $R_{j}$ the rotation matrix corresponding to the *j^th^* camera frame.

**Figure 8:**
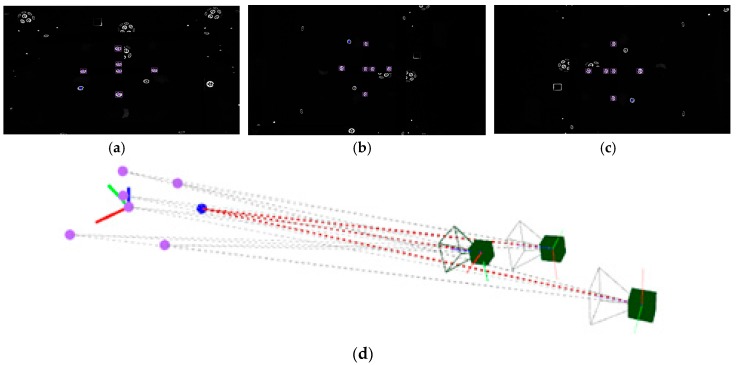
Computing of initial approach of a new target *X_i_* coordinates. A coded target is detected (highlighted in blue) in each image (**a**–**c**), where images corresponds to those three in Figure 7. Computed target coordinates are depicted in 3D scene (**d**) (in blue), along with the three epipolars from the images to the detected target.

Following the example in Section 4.1, given the computed extrinsic parameters of the set of three camera views (Table 2), the iteration of $∆\theta_{x_{i}}$ and the computation of the *X_i_* coordinates proceeds for coded targets detected at least in two camera views. Figure 8 shows an example of computation of a coded target detected in all three images (Figure 8a–c). Again, nominal values are adopted for the camera intrinsic parameters. Figure 8d depicts the 3D location computed for that new target in the same measuring frame as camera views are given. 

Along with computing the new target *X_i_* coordinates, new camera extrinsic parameters also enable code assignation to non-coded targets with pending correspondence to solve between detected coordinates in different images. The set of 3D epipolar lines given by the non-coded targets detected in all images can be expressed as 2D epipolar lines in each image. The distances from each 2D epipolar line to each non-coded target detected in the image can be calculated, so that the subset of 2D epipolar lines closest to a specific non-coded target in that image are likely to correspond to the epipolars of that 3D target detected in the other images. All possible 2D epipolar-to-target distances can be computed according to all possible correspondences between non-coded targets detected in three consecutive images, and the Hungarian method [24,29] can be adopted for solving the correspondences as the optimal combination which minimizes epipolar-to-target distance distribution among all 2D epipolars and non-coded target coordinates. As a result, codes can be assigned to non-coded targets in each image, and their 3D coordinate can be correspondingly computed.

**Figure 9:**
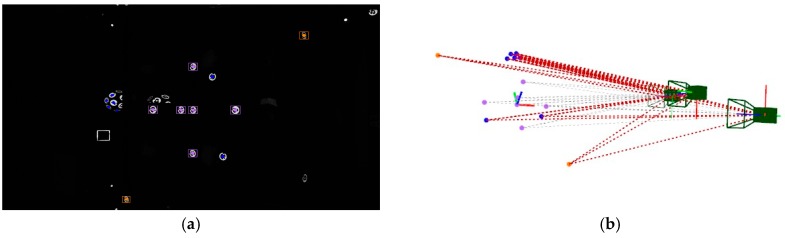
Example of target detection (**a**) in image 3 (Figure 8c), including coded targets (blue) and non-coded targets with solved correspondences (red), along with targets on the measuring reference frame (purple). 3D view of the scene (**b**) including the epipolar net (red) for solving the detected 3D target coordinates (blue and red) given by the three images in Figure 8.

Figure 9a shows all the detected targets in the image presented in Figure 8c, including both coded targets and non-coded targets with solved correspondences between the three images. Figure 9b shows the resulting scene including the 3D coordinates of the computed new targets, along with the 3D epipolar net used to solve them. Computed target coordinates are listed in Table 3, along with corresponding code identification and assignation (id) for coded and non-coded targets, respectively. The reprojection error minimization performance is also shown for computing each individual target, with a RMS value ranging from 0.085 pixels to 1.617 pixels. Again, the mean computing time ranges below 0.1 ms per iteration.

### 4.3. Joint Bundle Adjustment

Once the new target coordinates are solved, other than those defining the measuring frame, the bundle adjustment for joint computation (θ in Equation (7)) of *i =* 1 .. *N* target 3D coordinates ($\theta_{x_{i}}$) and *j* = 1 ..*M* camera extrinsic parameters ($\theta_{e_{j}}$) proceeds, given by their interdependency through the epipolar net multiple view geometry (Figure 6c). Now, partial derivatives (Equation (9)) in a Jacobian matrix J can be defined (Equation (19)) for minimizing a joint reprojection error vector of *2 m* elements, being m as defined at the end of Section 3.

(19) J(θ)=[E X]

with ${\left(E\right)}_{2m\times 6M}$ and ${\left(X\right)}_{2m\times 3N}$ containing the partial derivatives of each reprojection error with respect to all camera extrinsic (*α_j_*, *β_j_*, *γ_j_*, and *d_j_*) and target coordinates (*X_i_*), respectively. Reprojection error vector elements can be arranged so that E can be defined as a non-square diagonal matrix with ${\left(E_{j}\right)}_{2N_{j}\times 6}$ submatrices in its diagonal with the partial derivative to each image extrinsic parameters $\theta_{e_{j}}$ (Equation (11)) per the set of *N_j_* reprojection errors of all targets detected in each *j*-th image, and X can be correspondingly expressed as a sparse matrix with ${\left(X_{ij}\right)}_{2\times 3}$ submatrices with partial derivative to each target coordinate $\theta_{x_{i}}$ (Equation (17)) per the set of *M_i_* reprojection errors of each *i^th^* target detected in its subset of images per X column and zeros in the rest of elements. Given this arrangement, $J^{T}J$ in Equation (8) can be expressed as follows

(20) $${\left[E\;X\right]}^{T}\left[E\;X\right]=\left[\begin{array}{cc} A & B\\ B^{T} & C \end{array}\right]$$

where ${\left(A\right)}_{6M\times 6M}$ is a square diagonal matrix in which its diagonal is composed of ${\left(E_{j}{}^{T}E_{j}\right)}_{6\times 6}$ square submatrices accounting for each *j*-th camera extrinsic contribution to the minimization problem in the least square sense, given $E_{j}$ as defined in Equation (11), and ${\left(C\right)}_{3N\times 3N}$ is also a square diagonal matrix in which its diagonal is composed of ${\left(\overset{M_{i}}{\underset{j=1}{\sum }}X_{ij}^{T}X_{ij}\right)}_{3\times 3}$ square submatrices accounting for the contribution of the 3D coordinate of each corresponding *i*-th target detected in its *M_i_* image subtset, given $X_{ij}$ as defined in Equation (17).

${\left(B\right)}_{6M\times 3N}$ is a non-square sparse matrix where the interdependency between extrinsic parameters and target coordinate computation through the joint epipolar net is taken into account, with elements ${\left(E_{ij}^{T}X_{ij}\right)}_{6\times 3}$ contributing along with the joint product of the partial derivatives to image extrinsic and target coordinates for a *i*-th target detected in a *j*-th image, and zero when a *i*-th target is not seen in a *j*-th image, given $E_{ij}$ as defined in Equation (12).

Thus, according to Equation (20), Equation (8) can be decomposed as

(21) $$\left[\begin{array}{cc} A & B\\ B^{T} & C \end{array}\right]\left[\begin{array}{c} {∆}_{\theta_{e}}\\ {∆}_{\theta_{x}} \end{array}\right]=-J^{T}r$$

where the residual term $J^{T}r$ can be redefined according to Equation (19) as

(22) $$\left[\begin{array}{c} \epsilon_{e}\\ \epsilon_{x} \end{array}\right]=-J^{T}r=-\left[\begin{array}{c} E^{T}\\ X^{T} \end{array}\right]r$$

Given Equations (21) and (22), the linear system in Equation (8) can now be decomposed to a set of two lower range subsystems where ${∆}_{\theta_{e}}$ and ${∆}_{\theta_{x}}$ can be individually solved for interdependent extrinsic and target coordinate iteration, where extrinsic iteration ${∆}_{\theta_{e}}$ can be obtained as

(23) $${∆}_{\theta_{e}}={\left(A-BC^{-1}B^{T}\right)}^{-1}\left(\epsilon_{e}-BC^{-1}\;\epsilon_{x}\right)$$

and target coordinate iteration ${∆}_{\theta_{x}}$ can correspondingly be given as

(24) $${∆}_{\theta_{x}}=C^{-1}\left(\epsilon_{x}-B^{T}\;{∆}_{\theta_{e}}\right)$$

Following the examples in Section 4.1 and Section 4.2, given the computed initial approaches for the three first image extrinsic parameters (Table 2) and for the corresponding target coordinates (Table 3) in the evaluation scene shown in Figure 9b, their joint computation can be conducted according to Equations (23) and (24). As inputs for the method, 3D coordinates of the set of coded targets on the measuring frame are known (Table 1), as well as nominal camera intrinsic parameters, along with image coordinates of coded and non-coded target with solved correspondences between images (Table 1 and Table 3). Table 4 and Table 5 show the results of the intermediate joint bundle computing for extrinsic parameters and targets (step 6 of the in-process procedure), respectively. The RMS of the joint reprojection error is optimized to 0.802 pixel, with an iteration time of 0.17 ms.

**Figure 10:**
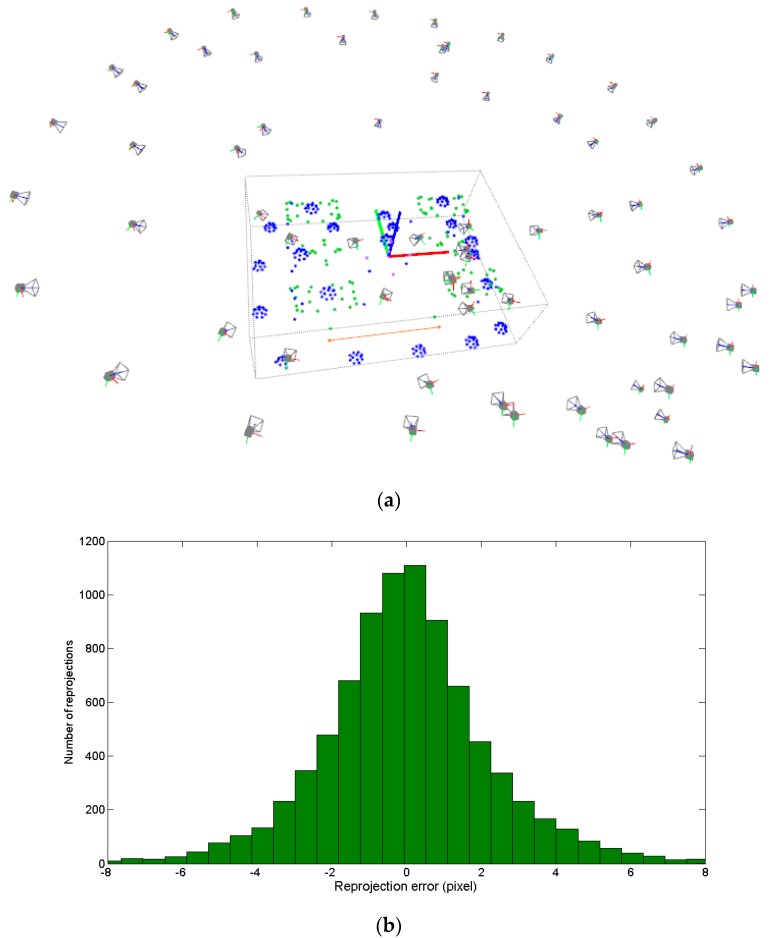
Measurement results for the pilot case (Figure 3) after bundle adjustment (**a**), where camera frames and the set of measured targets (coded in blue, non-coded in green) are shown given their computed coordinates in the reference frame (blue, green, and red axes) along with a scale bar for precise scale definition (orange), and histogram showing the reprojection error vector distribution in pixels (**b**) after including the scale bar distance (orange) into the joint bundle.

The measuring process may now continue with the acquisition and in-process computing of new images, following the in-process procedure described in the introduction of Section 4 (steps 1 to 6). An example of a complete measuring set is shown in Figure 10a for the pilot case under study (Figure 3), where non-coded targets were placed on the milled surfaces and coded targets were placed around the scene enabling a consistent epipolar net construction during measurement, along with the reference frame with its corresponding coded targets. A total number of 68 images were taken, solving the 3D coordinates of 80 non-coded (20 at each milled prism) and 340 coded targets. A maximum joint bundle computation time ranging 150 ms was observed for the last images of the measuring process (see Section 4.4).

So far, the measuring scale and traceability depend on the coded target 3D coordinates defining the reference frame (Table 1), along with the adopted nominal camera intrinsic parameters. An alternative for increasing accuracy in portable photogrammetry is the adoption of appropriate scale bars with calibrated lengths between corresponding pairs of optical targets (highlighted in orange in Figure 10a), so that measuring process traceability is given by precise scale bar distances. Then, once image-taking finishes (steps 1 to 6), the final post-process joint bundle of camera extrinsic parameters and target coordinates can proceed until convergence (step 7), imposing calibrated relative distances between corresponding pairs of target coordinates in each scale bar available in the scene. A free network adjustment is then carried out. For this, the 3D coordinates of the coded targets on the reference frame (Table 1) have to be also computed, so that their assumed coordinates so far (in steps 1 to 6) do not influence measuring process traceability, and the measuring frame is correspondingly redefined according to their computed coordinates following the widely adopted 3 (to define a reference plane) −2 (to define a reference axis) −1 (to define the origin point) rule in metrology, setting back a determined origin and orientation to the measuring coordinate system.

To do so, a corresponding error vector $r_{t}$ can be expressed (Equation (25)) and its corresponding joint minimization can be accomplished along with the reprojection error vector r in Equation (4).

(25) $${\left(r_{t}\right)}_{\left(B+6\right)\times 1}=\left[\begin{array}{c} r_{b}\\ r_{x} \end{array}\right]$$

where $r_{b}$ expresses (Equation (26)) the square distance between each pair of target coordinates minus the corresponding scale square length, given by ${\left|x_{k}^{b}-x_{k}^{e}\right|}^{2}-b_{k}^{2}$, from *k = 1 ..B* bars with $b_{k}^{}$ scales in the scene, with $x_{k}^{b}$ and $x_{k}^{e}$ being the 3D computed coordinates for each pair of targets of the *k^th^* scale bar, which can be expressed as

(26) $${\left(r_{b}\right)}_{B\times 1}=\left[\begin{array}{c} \begin{array}{c} {\left|x_{1}^{b}-x_{1}^{e}\right|}^{2}-b_{1}^{2}\\ \vdots \end{array}\\ \begin{array}{c} {\left|x_{k}^{b}-x_{k}^{e}\right|}^{2}-b_{k}^{2}\\ \vdots \end{array}\\ {\left|x_{B}^{b}-x_{B}^{e}\right|}^{2}-b_{B}^{2} \end{array}\right]$$

and $r_{x}$ contains (Equation (27)) the 3D coordinate errors of a set of reference targets selected for determining the measuring coordinate system according to the 3-2-1 rule, which can be expressed as

(27) $${\left(r_{x}\right)}_{6\times 1}={\left[x_{0}\;y_{0}\;z_{0}\;y_{1}\;z_{1}\;z_{2}\right]}^{T}$$

where a first reference target *X_0_* is set and constrained to be the coordinate system origin (X_1_ in Table 1), so that $X_{0}={\left[x_{0}\;y_{0}\;z_{0}\right]}^{T}$ coordinates have to be minimized to zero as first elements in $r_{x}$ setting 3 restrictions, a second reference target X_1_ is constrained (X_3_ in Table 1) to have its *Y* and *Z* coordinates to zero, so that $y_{1}$ and $z_{1}$ coordinates in $X_{1}={\left[x_{1}\;y_{1}\;z_{1}\right]}^{T}$ are included as the next elements in $r_{x}$ setting 2 restrictions determining *X* coordinate axis, and a last reference target *X*_2_ is constrained (*X*_4_ in Table 1) to have its *Z* coordinate to zero, so that $z_{2}$ coordinate in $X_{2}={\left[x_{2}\;y_{2}\;z_{2}\right]}^{T}$ is included as the last elements in $r_{x}$ setting 1 restriction determining *XY* plane of the measuring frame. As a result, 6 constraints are included and a coordinate system is determined, the rest of the initially assumed reference target coordinates (Table 1) being unconstrained and correspondingly computed, so that measuring traceability is now set by the scales imposed in Equation (26).

A Jacobian matrix G corresponding to $r_{t}$ minimization problem (as in Equation (8)) can be defined as follows

(28) $${\left(G\right)}_{\left(B+6\right)\times \left(3N\right)}=\left[\begin{array}{c} G_{b}\\ G_{x} \end{array}\right]$$

where ${\left(G_{b}\right)}_{B\times \left(3N\right)}$ contains (Equation (29)) the partial derivatives of the *k* = 1 ..*B* square distance errors to the each pair of corresponding target coordinates $x_{k}^{b}$ and $x_{k}^{e}$ given as

(29) $${\left(G_{b}\right)}_{B\times \left(3N\right)}=\left[\begin{array}{c} G_{1}\\ \vdots \\ \begin{array}{c} \begin{array}{c} G_{k}\\ \vdots \end{array}\\ G_{B} \end{array} \end{array}\right]$$

being each $G_{k}$

(30) $${\left(G_{k}\right)}_{1\times \left(3N\right)}=\left[\cdots \;\cdots \;\cdots \;Dx_{k}^{b}\;\cdots \;\cdots \;\cdots \;Dx_{k}^{e}\;\cdots \;\cdots \;\cdots \right]$$

with derivatives $Dx_{k}^{b}$ and $Dx_{k}^{e}$ following Equations (31) and (32) and zeros for the rest of the elements in $G_{k}$, being *N* the new total number of computed targets,

(31) $$Dx_{k}^{b}=2\left[\left(x_{k}^{b}-x_{k}^{e}\right)\;\left(y_{k}^{b}-y_{k}^{e}\right)\;\left(z_{k}^{b}-z_{k}^{e}\right)\right]$$

(32) $$Dx_{k}^{e}=-Dx_{k}^{b}$$

and ${\left(G_{x}\right)}_{6\times \left(3N\right)}$ expresses the partial derivatives of $r_{x}$ to the constrained reference target coordinates, equal to 1 at the columns corresponding to each computed coordinate, and zeros in the rest of the elements. 

Thus, a joint error vector $r_{s}$ can be defined as follows (Equation (33)) integrating reprojection errors in r along with error vector $r_{t}$

(33) $$r_{s}=\left[\begin{array}{c} r\\ r_{t} \end{array}\right]$$

and joint bundle can be conducted (as in Equation (8)) with the corresponding redefined joint Jacobian for numerical iteration as

(34) $${\left(J\right)}_{\left(2m+B+6\right)\times \left(6M+3N\right)}=\left[\begin{array}{cc} E & X\\ 0 & G \end{array}\right]$$

Following the same system decomposition approach as in Equations (21)–(24), it can be demonstrated that the main iteration equations given by Equation (21) can be redefined to a similar form as

(35) $$\left[\begin{array}{cc} A & B\\ B^{T} & C_{t} \end{array}\right]\left[\begin{array}{c} {∆}_{\theta_{e}}\\ {∆}_{\theta_{x}} \end{array}\right]=\left[\begin{array}{c} \epsilon_{e}\\ \epsilon_{x_{t}} \end{array}\right]$$

where terms C and $\epsilon_{x}$ are redefined to $C_{t}$ and $\epsilon_{x_{t}}$ according to G and $r_{t}$ as

(36) $$C_{t}=C+G^{T}G\;\epsilon_{x_{t}}=\epsilon_{x}-G^{T}r_{t}$$

so that expressions given at Equations (23) and (24) for ${∆}_{\theta_{e}}$ and ${∆}_{\theta_{x}}$, respectively, can be used for computing to convergence the post-process joint bundle (step 7) of the scene extrinsic and target coordinates integrating scale and 3-2-1 rule restrictions. As input for the post-process joint bundle, results after processing the last image at step 6 are known. Figure 10b shows the corresponding histogram of the minimized reprojection error vector r after joint bundle with $r_{t}$, resulting in a RMS value of 2.378 pixel. 

### 4.4. Computing Performance of the In-Process Approach

The procedure described above was developed in C++ language, using the OpenCV library for image processing and the Eigen library for matrix management and processing, on a desktop PC Intel Core i7-5600U 2.6 Ghz, with 16 Gb RAM, running on Windows 7 with 64 bits. Wireless image transmission was used, observing a transmission time of 0.5–1 s per image for 12.2 Mpixel raw images. Image processing time (step 1) was observed to reach 0.5 s per image, depending on the number of segmented and decoded targets on the image. Computing time for the initial extrinsic and target approach (steps 2 and 5) for new images and targets prior to their first bundle could be relatively neglected, ranging below 1 ms. Code assignation to non-coded targets (step 4) was observed to range up to 0.1–0.3 s per image, depending on the number of non-coded targets and correspondences to solve between images.

**Figure 11:**
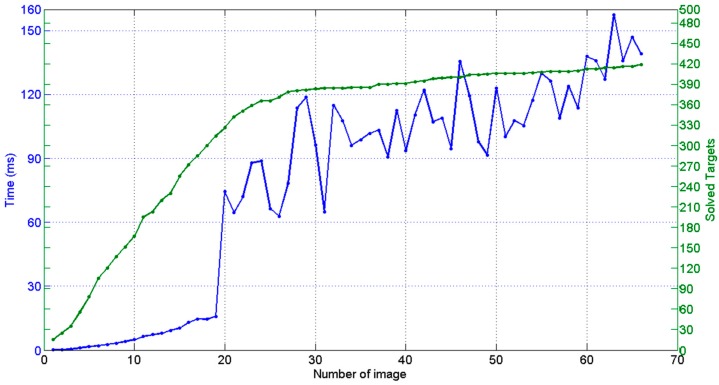
In-process intermediate bundle (step 6) computing time (ms) and number of solved targets each time a new image is taken during the measuring process of the scenario depicted in Figure 10. A discontinuity is observed at image 20 bundle computing time, which corresponded to a steep computational change in the management and allocation time (Eigen library) of submatrices in Equation (23).

Figure 11 shows the dependence of the computation time of the intermediate bundle (step 6) on the number of cumulated images and solved targets so far during the measuring process, with a time ranging up to 0.15 s per image for the last images of the measuring scenario shown in Figure 10. As a result, total in-process computation time (steps 1 to 6) ranged at a maximum of 1 s per image, in the same order of magnitude of the wireless image transmission time itself.

However, an average increase of 3 ms per additional image can be estimated from Figure 11 for step 6, which would lead to relevant in-process computation time contribution if larger measuring scenarios were adopted with more images and targets. If a better in-process computing performance was required for step 6, further optimization could be conducted following the system decomposition approach presented in this paper, taking advantage of the symmetry and characteristics of the involved submatrices in Equation (21) (diagonal A and C submatrices, dominating presence of zeros in sparse B submatrix, etc.) along with the development of analytic expressions for the involved inverse matrices in Equations (23) and (24).

Finally, the post-process joint computation time (step 7) reached 3 s in the same scenario, with 5 iterations to convergence, assuming convergence criteria of ${∆}_{\theta_{e}}$ and ${∆}_{\theta_{x}}$ being below a minimum iteration value of 10^−6^ mm and 10^−6^ radians for all location and orientations, respectively.

## 5. Camera Model Self-Calibration for Precision

**Figure 12:**
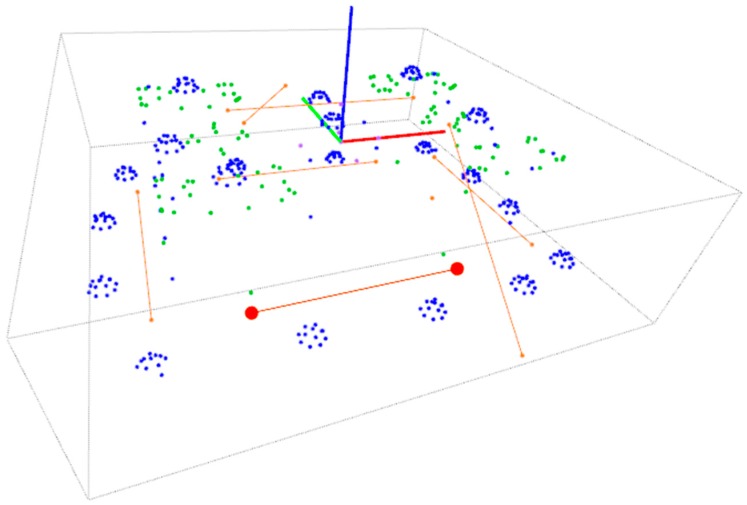
LME approach for precision evaluation, with one calibrated bar as the scale for measurement (red, same scale as in Figure 10a and L3 in Figure 3) and six bars for length error evaluation (orange), all covering the measuring scene of the pilot case under study, with multicoded artifacts (in blue, as shown previously in Figure 2 and Figure 3) and non-coded targets on prismatic subelements (green).

Along with scale bars in the scene, camera intrinsic parameters determine measuring process traceability. Nominal camera intrinsic parameters have been adopted for the results shown so far. As introduced in Section 2, a length measuring error (LME) approach similar to VDI 2634 can be adopted for evaluating measuring procedure uncertainty in portable photogrammetry. Figure 12 shows the same measuring scenario as in Figure 10a, but additional six scale bars are included covering the scene so that just one bar is used for imposing scale to the measurement and LME errors can be evaluated on the rest of measured scales.

A maximum LME error of 1.2 mm was observed in the photogrammetric measurement given calibrated scale lengths ranging 1340 mm, corresponding to a limited relative precision of approximately 1/1000 similar to the reported in [5], one order of magnitude below the expected performance of portable photogrammetry using precise pre-calibrated cameras, where a relative precision ranging better than 1/10,000 is typically reported [20] for 1 m long scenes.

Other than assuming nominal values for intrinsic parameters, precise camera calibration is required for precise measuring. Accurate camera calibration is necessary in photogrammetry systems and that is why continuous and recent improvements regarding mathematical analysis of the data taken for self-calibration [11,12,13] and image distortion correction [14,15] can be found in the literature. Nowadays there are several software applications for close range photogrammetry in industry using optical targets (i.e., Aicon 3D, Geodetic V-Stars, Creaform MaxShot3D, GOM Tritop, etc.) that can automatically perform camera self-calibration. These photogrammetry specific commercial systems present a main limitation when talking about their self-calibration since they only provide this option by a specific procedure that must be performed out of the measuring process itself since traditional self-calibration methods need precise calibration pattern and extensive post-processing work. This does not enable the option of in-process self-calibration. Such self-calibration capabilities integrated into the measuring process would be essential to compensate for main uncertainty contributors, especially by determining the intrinsic parameters of the camera model on the fly. In addition, such a self-calibrated machine vision would enable the use of low-cost cameras—and not only specific photogrammetry cameras—for metrology applications. 

Some approaches for the calibration of commercial digital cameras in different applications can be found in the literature. In [32] an accuracy comparison is carried out between three low-cost consumer grade digital cameras and a specific photogrammetry proven camera. In [33] a study of different commercial software options for the calibration and self-calibration of cameras mounted on unmanned aerial vehicles (UAVs) is presented. A similar approach for the self-calibration of commercial small action cameras applied to photogrammetric purpose is proposed in [34] by using a 2D external calibrated reference and a specifically developed software. However, the options presented do not allow in-process self-calibration since they need extensive post processing. 

To overcome the time limitations of off-process calibrations, some approaches propose diagnosis methods for camera internal parameters in order to prevent the need to stop the measuring process so often to check them: in [35] a diagnostic method for internal parameters based on multivariate control charts is proposed in order to provide a comprehensive stability control over all the performed calibrations for systems used for regular monitoring of production lines. However, this cannot be considered a self-calibration, but a stability control of the intrinsic parameters.

Special attention should be paid to texture-based software for 3D reconstruction and modelling (i.e., Agisoft PhotoScan, Photomodeler Scanner, Bundler, Pix4Dmapper, VisualSFM, iWitness, MicMac, 3DF Zephir, etc.), with a broad range of applications (e.g., mapping, architecture, mining, construction, agriculture, heritage recording, archeology, forensic 3D modelling, etc.). Other than using optical retroreflective targets, measuring results are obtained on object surface textures available in the images. Texture-based techniques (SIFT [36] and SURF [37] image descriptors, SfM [38,39,40], etc.) are used, enabling dense 3D surface meshing and reconstruction. In-process camera model self-calibration techniques are integrated, demonstrating the capability of 3D surface reconstruction and modelling using consumer grade low-cost cameras [41,42,43]. However, strong limitations can be found for taking these texture-based approaches to precise industrial metrology. Textures available in the images depend on object characteristics and light conditions, limiting measurement precision and reliability compared to the use of physical optical targets. Additionally, computationally hard texture-based approaches are required, with, again, extensive post-processing times, limiting its application for efficient measuring processes in industry by photogrammetry. 

The in-process self-calibration method proposed in this work overcomes these limitations by allowing its computationally efficient application for precise industrial metrology applications using optical targets. The method for the self-calibration of camera and lens distortion has been integrated due to its potential for highest precision and accuracy level [20,44] when using low cost non specialized digital cameras, where the solved 3D point cloud scene itself is used as calibration geometry. Measuring traceability is set only by the scale bars in the measuring scene, avoiding uncertainty contributors from off-process camera calibration processes. The camera can be continuously controlled and compensated on the fly, bringing the potential of getting independent measurement results to changes in camera condition over time, such as with thermo-mechanically unstable low-cost cameras. As a step forward to off-process camera calibration and in-process stability control, the approach adopted in this work consist in taking advantage of redundant information available in the portable photogrammetry scene, so that, along with extrinsic and target coordinates, camera intrinsic parameters are also included into a final bundle adjustment computing, having as the only inputs the measuring images themselves and the scale bar distances for precise traceability.

### 5.1. Including Camera Model into Bundle Adjustment

Computation of the camera intrinsic parameters means determining vector $\theta_{i}$ (Equation (37)) along with camera extrinsic $\theta_{e}$ (Equation (5)) and target coordinates in $\theta_{x}$ (Equation (6)) in a joint bundle minimizing error vector $r_{s}$ (Equation (33)) including scale bars restrictions.

(37) $${\left(\theta_{i}\right)}_{7\times 1}={\left[f\;\;h_{0}\;v_{0}\;k_{1}\;k_{2}\;\pi_{1}\;\pi_{2}\;\right]}^{T}$$

where the focal distance f, the coordinates of the distortion center ($h_{0}$*,*$v_{0}$), the radial distortion coefficients ($k_{1}$ and $k_{2}$) and the tangential distortion coefficients ($\pi_{1}$ and $\pi_{2}$) are included.

Hence, θ vector to compute in the joint bundle is redefined as

(38) $$\theta =\left[\begin{array}{c} \theta_{e}\\ \theta_{x}\\ \theta_{i} \end{array}\right]$$

and following Equation (34), a corresponding complete joint Jacobian matrix can be expressed as

(39) $${\left(J\right)}_{\left(2m+B+6\right)\times \left(6M+3N+7\right)}=\left[\begin{array}{ccc} E & X & F\\ 0 & G & 0 \end{array}\right]$$

where F expresses the partial derivatives of the reprojection error vector r to the camera intrinsic parameters in $\theta_{i}$, so that

(40) $${\left(F\right)}_{2m\times 7}=\left[\begin{array}{c} F_{11}\\ \vdots \\ \begin{array}{c} \begin{array}{c} F_{ij}\\ \vdots \end{array}\\ F_{2m} \end{array} \end{array}\right]$$

being $F_{ij}$ the partial derivatives of the reprojection error $r_{ij}$ of the *i*-th target in the *j*-th camera view, according to the projection model in Equation (2) and the distortion model in Equation (3), as

(41) $$F_{ij}=\left[\begin{array}{ccccccc} \frac{\partial r_{ij}}{\partial f} & \frac{\partial r_{ij}}{\partial h_{0}} & \frac{\partial r_{ij}}{\partial v_{0}} & \frac{\partial r_{ij}}{\partial k_{1}} & \frac{\partial r_{ij}}{\partial k_{2}} & \frac{\partial r_{ij}}{\partial \pi_{1}} & \frac{\partial r_{ij}}{\partial \pi_{2}} \end{array}\right]$$

Given the partial derivatives in F and following again the same system decomposition approach as in Equation (35) for the join bundle including scale bars, the main iteration equations including camera self-calibration can be redefined as

(42) $$\left[\begin{array}{ccc} A & B & E_{f}\\ B^{T} & C_{t} & D_{f}\\ E_{f}^{T} & D_{f}^{T} & F_{f} \end{array}\right]\left[\begin{array}{c} {∆}_{\theta_{e}}\\ {∆}_{\theta_{x}}\\ {∆}_{\theta_{f}} \end{array}\right]=\left[\begin{array}{c} \epsilon_{e}\\ \epsilon_{x_{t}}\\ \epsilon_{f} \end{array}\right]$$

where

(43) $$F_{f}=F^{T}F$$

(44) $$E_{f}=E^{T}F$$

(45) $$D_{f}=X^{T}F$$

being F, E and X defined as in Equations (40) and (19), respectively, where ${\left(F_{f}\right)}_{7\times 7}$ is a square symmetric matrix accounting for each camera intrinsic parameter direct contribution to the minimization problem, and ${\left(E_{f}\right)}_{6M\times 7}$ and ${\left(D_{f}\right)}_{3N\times 7}$ are non-square matrices accounting for the corresponding joint product of partial derivatives integrating the interdependency between extrinsic and target coordinate computing with camera intrinsic parameters through the joint epipolar net, respectively, and $\epsilon_{f}$ is expressed as

(46) $$\epsilon_{f}=F^{T}r$$

analog to $\epsilon_{e}$ and $\epsilon_{x}$ in Equation (22).

From the linear system in Equation (42), it can be obtained

(47) $${∆}_{\theta_{f}}=F_{f}{}^{-1}\left(\epsilon_{f}-E_{f}{}^{T}{∆}_{\theta_{e}}-D_{f}{}^{T}{∆}_{\theta_{x}}\right)$$

and then, Equation (42) can be reduced to a similar system as Equation (35)

(48) $$\left[\begin{array}{cc} A_{f} & B_{f1}\\ B_{f2} & C_{f} \end{array}\right]\left[\begin{array}{c} {∆}_{\theta_{e}}\\ {∆}_{\theta_{x}} \end{array}\right]=\left[\begin{array}{c} \epsilon_{e_{f}}\\ \epsilon_{x_{f}} \end{array}\right]$$

where ${∆}_{\theta_{e}}$ and ${∆}_{\theta_{x}}$ can be obtained following analog equations to Equations (23) and (24), substituting A, B, $B^{T}$, C, $\epsilon_{e}$ and $\epsilon_{x}$, by $A_{f}$, $B_{f1}$, $B_{f2}$, $C_{f}$, $\epsilon_{e_{f}}$ and $\epsilon_{x_{f}}$, given them as

(49) $$A_{f}=A-E_{f}F_{f}{}^{-1}E_{f}{}^{T}$$

(50) $$B_{f1}=B-E_{f}F_{f}{}^{-1}D_{f}{}^{T}$$

(51) $$B_{f2}=B^{T}-D_{f}F_{f}{}^{-1}E_{f}{}^{T}$$

(52) $$C_{f}=C_{t}-D_{f}F_{f}{}^{-1}D_{f}{}^{T}$$

(53) $$\epsilon_{e_{f}}=\epsilon_{e}-E_{f}F_{f}{}^{-1}\epsilon_{f}$$

(54) $$\epsilon_{x_{f}}=\epsilon_{x_{t}}-D_{f}F_{f}{}^{-1}\epsilon_{f}$$

### 5.2. Computing Efficiency and Precision Performance Evaluation for Self-Calibrated Photogrammetry

Bundle computing that includes self-calibration can be conducted following Equations (47) and (48) for the joint iteration of $\theta_{e}$, $\theta_{x}$, and $\theta_{i}$ to convergence, having as initial approach the previous joint bundle computation given constant camera intrinsic parameters. Following the examples in Figure 10 and Figure 12, post-process joint bundle including self-calibration was conducted including the same scale bar (L3) for traceability. 

**Figure 13:**
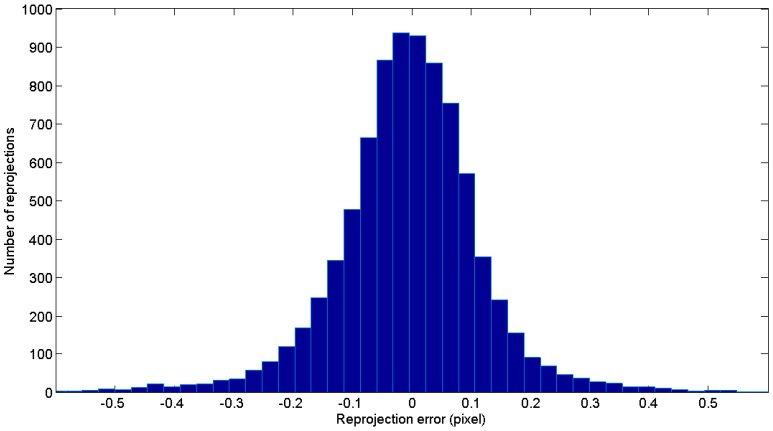
Histogram of reprojection error vector after self-calibration in pixels, including one scale bar distance for traceability (Figure 10, in orange), with RMS value being 0.18 pixel.

Figure 13 shows the histogram of the optimized reprojection error distribution, where an order of magnitude lower RMS value is obtained compared to that obtained by assuming nominal camera instrinsics (histogram in Figure 10b). Table 6 shows the computed camera intrinsic parameters for this example, optimized to those assumed to be nominal in Section 4. Post-process joint computation including self-calibration took up to 8 s, with 11 iterations to convergence. As a result, the total post-processing after measuring was limited to 8 + 3 = 11 s, enabling on the fly camera self-calibration and measuring self-compensation, assuming a constant camera condition during measurement.

Again, the length measuring error (LME) approach was adopted for evaluating measuring uncertainty in self-calibrated portable photogrammetry. A set of 10 consecutive photogrammetric measurements were taken on the scene. Each measurement was solved imposing L3 scale bar (Figure 12), and remaining scales were used as control bars for LME evaluation. Maximum LME between targets on control scale bars obtained was 121.4 μm in L0, with a standard deviation for all the set of measurements of 45.1 μm. However, except for L0, the values obtained showed relatively lower LME results for lengths measured in the *XY* plane (ranging below 40 μm). Nevertheless, that maximum observed LME was assumed as a conservative estimation to characterize optical target measurement process uncertainty in all spatial directions, corresponding to a relative precision ranging 1/10,000.

**Figure 14:**
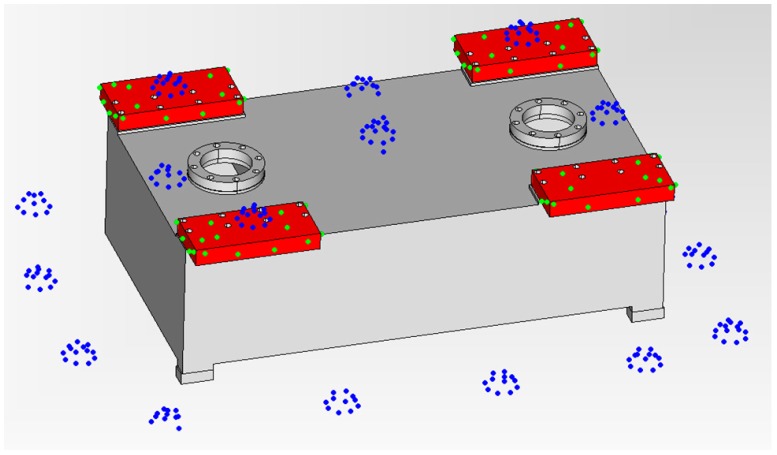
Fitting of non-coded target coordinates (green) to nominal reference surfaces on CAD file (red) for evaluating measuring error, along with auxiliary multicoded targets in the scene (blue).

According to the maximum observed LME value of 121.4 μm, the spatial uncertainty for the target coordinates can be estimated in 70.1 μm (σ), given that σ=LME/√3 assuming a rectangular distribution. As an alternative way of evaluating measuring performance, the 3D coordinates of the computed non-coded targets in each measurement, properly placed on relatively precise milled prismatic elements of the pilot case (see Figure 3), can be fitted and compared with its nominal geometry [5], and the corresponding errors to each nominal surface can be evaluated for all targets in all 10 measurements. Figure 14 shows an example of fitting of a measurement result to the nominal reference geometry for error evaluation. A maximum standard deviation (σ) of 14 μm was observed for the measuring errors of non-coded targets to all surfaces in the *XY* plane in all measurements, but up to 60 μm for the measuring errors of the targets laying in lateral surfaces, close to the estimated σ of 70.1 μm given by the LME evaluation. This result points out again the conservative character of the spatial uncertainty (σ) value previously estimated by the LME approach, due to the observed anisotropy on the non-coded target measurement accuracy. In any case, a relative precision ranging 1/10,000 by LME analysis is confirmed in the worst-case scenario for all spatial directions, demonstrating the adequate accuracy of the developed in-process self-calibrated photogrammetry in the pilot case scenario, comparing to previously reported limited performance of 1/1000 relative precision [5], ranging now at the same precision level as the performance expected when using precise pre-process camera calibration.

## 6. Evaluation at Industrial Scenarios

**Figure 15:**
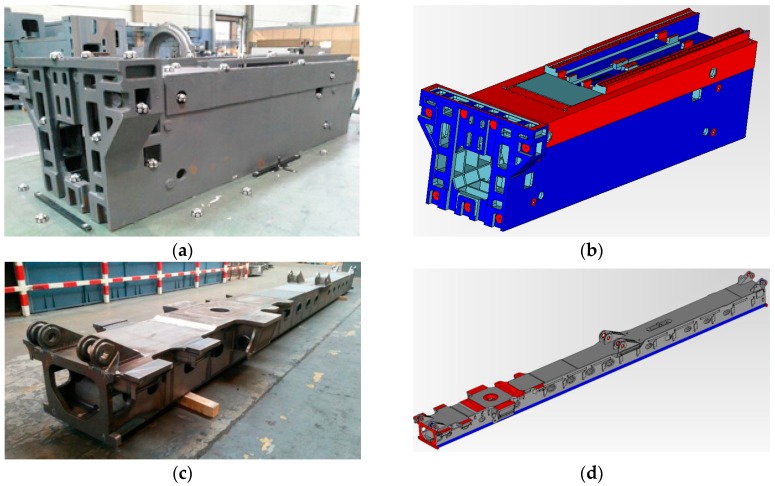
Evaluation tests at industrial scenarios for two end-users (Goimek and Liebherr). Large raw parts prior to their machining are shown, (**a**) Soraluce milling machine travelling column (6 m long) manufactured at Goimek machining shop, and (**c**) Liebherr drilling rig lead center (15 m long), along with corresponding CAD views (**b**,**d**) showing measured surfaces (in red) for overstock control.

Finally, the self-calibrated in-process photogrammetry system has been evaluated in an industrial scenario with two different parts (Figure 15a,c) demonstrating a fast, reliable and precise raw part measuring process. The system was applied for the measurement and alignment of up to 15 m long raw parts prior to their machining, at least one order of magnitude larger than the considered pilot case (Figure 3), with scene sizes ranging now up to 200 m^3^.

**Figure 16:**
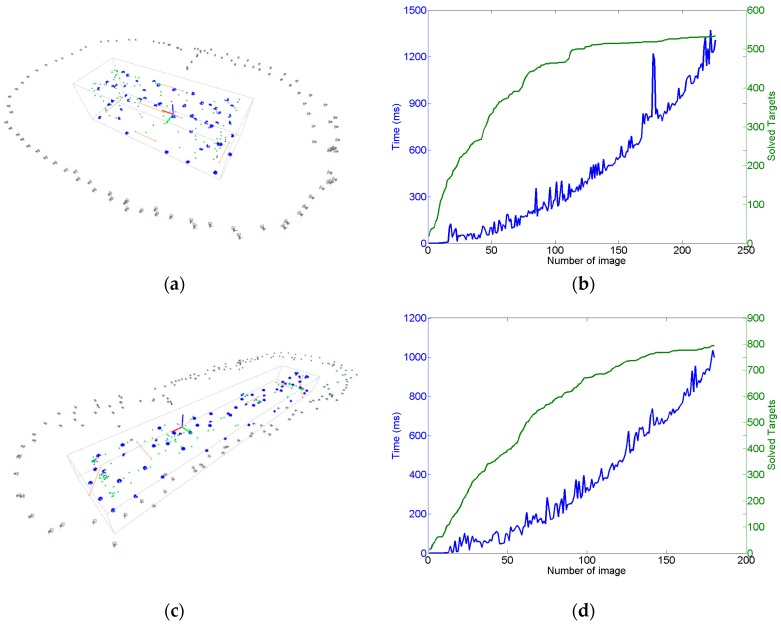
Measurement results (**a**,**c**) in large raw parts for industrial examples shown in Figure 15a,c respectively, where solved target 3D coordinates and camera views are jointly depicted, and corresponding in-process intermediate bundle computing times (**b**,**d**).

The measurement process took an overall time of 1 h per part in both industrial scenarios, determining the 3D coordinates of the set of non-coded target placed on the surfaces to be machined. An approximated total number of 800 targets and 200 images were computed in both scenarios (Figure 16a,c). Performance of the in-process computing procedure was observed, ranging now at a total maximum in-process computing time of almost 2 s per image for the last images taken during the measuring processes, higher than the 1 s per image observed at the pilot case scenario, but still enabling practical quasi real time diagnosis and control of correct images taken in an industrial scenario, so that every time an incorrect images was acquired (due to an image not contributing to a consistent epipolar net, pending target to be solved in a particular zone of the scene, etc.) measuring could be properly guided to locally take new adequate images if necessary before continuing with the process.

The higher intermediate bundle computing time was the main contributor to the increase of the in-process computation time in the last stages of the measuring process, as expected by the dependence of the bundle computational work on the number of images and targets to solve (as shown in Figure 11). Along with the alternatives pointed out in Section 4.4 for increasing its computing performance, a strategy could also be adopted for limiting bundle adjustment only to a minimum subset of the scene measured so far, so that continuous computing of the joint bundle of the complete scene could be avoided or only periodically executed.

Accordingly, the post-process computing time including self-calibration of camera intrinsic parameters ranged a maximum of 15 s, slightly higher than the observed one in the pilot case scenario as a result of the higher number of images and targets, but meeting efficient operation at an industrial scenario.

Regarding precision, according to the LME evaluation results reported in the literature [20], typical scale dependent LME errors could be estimated as 50 μm + 20 μm/m for portable photogrammetry with precise pre-calibrated cameras. For the considered industrial cases, with up to 15 m long parts, an estimated LME performance of 0.35 mm could be expected as a reference for precise operation, corresponding to 0.20 mm (σ) spatial uncertainty for the measured targets, given that σ=LME/√3 assuming again a rectangular distribution.

**Figure 17:**
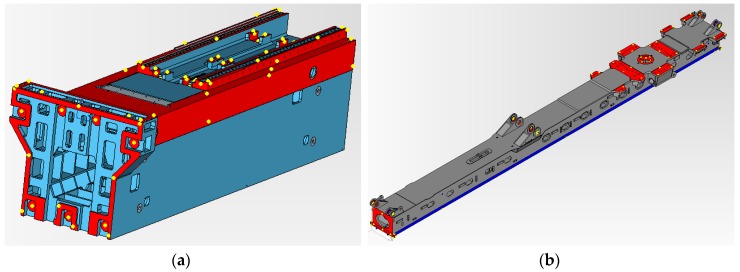
Fitting of measured non-coded targets (yellow) to the nominal part geometry, corresponding to the industrial examples ((**a**) Soraluce milling machine travelling column, and (**b**) Liebherr drilling rig lead center), where optimal target coordinates are given in the ideal part frame for an even overstock distribution in all surfaces to be machined (red).

After raw part measurement by photogrammetry, the complete process (Figure 1) was conducted towards in-machine raw part alignment in both industrial scenarios, so that gauging on the optical targets by a touch probe integrated in the machine (with machine axes typically ranging at 0.01 mm accuracy) was adopted as a reference for evaluating measuring precision. Optimal target coordinates were computed by fitting (Figure 17). Positive and even overstock was applied as in [5] as best-fitting criteria. A subset of targets was used as control points for alignment by a machine-integrated contact probe. In-machine fixturing of the part was adjusted to properly align it to machine axes so that probed relative coordinates between reference targets matched those optimally computed by fitting, to a difference below 0.1 mm so that alignment process uncertainty could be considered relatively neglected comparing to the measuring uncertainty of 0.2 mm (σ) estimated for the portable photogrammetry.

Once the part was precisely aligned, the measuring process performance was evaluated by contact probe gauging of a minimum set of 10 check points (i.e., non-coded targets not used as a reference for alignment), distributed in all 3 machine coordinate axes directions and at extreme and opposite surfaces of the part. A maximum probing error interval of ±0.6 mm (3σ) could be expected according to the above estimated target spatial uncertainty by LME (σ = 0.20 mm). Probing errors were evaluated between the overstock values resulting from the fitting process and the actual ones by the in-machine gauging to nominal surface coordinates. In both industrial scenarios (Figure 15a,c), all the probing errors ranged below ±0.5 mm, demonstrating the adequate accuracy of the developed self-calibrated in-process photogrammetry for large raw part measuring and overstock control.

## 7. Conclusions

A new efficient procedure has been presented in this work for solving the bundle adjustment problem in portable photogrammetry. In-process bundle computing capability is demonstrated on a consumer grade desktop PC, enabling quasi real time 2D image and 3D scene computing and diagnosis so that a reliable measuring procedure can be conducted, avoiding inefficient user-dependent post-process iterative procedures that limit the potential of portable photogrammetry for an easy, low-cost and fast solution for industrial metrology of large components.

A method for the self-calibration of camera and lens distortion has been integrated into the in-process approach due to its potential for highest precision and accuracy levels when using a non-specialized consumer grade digital camera (i.e., Nikon D300S), where the solved 3D point cloud scene itself is used as calibration geometry and measurement traceability is set only by the scale bars in the measuring scene, avoiding the need of off-process pre-calibrated cameras or the use of special purpose calibration artifacts in the scene for precise measurement in an industrial metrology application.

The developed self-calibrated in-process photogrammetry has been evaluated in a pilot case scenario (1.5 m long reference part) and at two large scale industrial scenarios (up to 15 m) for raw part measurement and alignment before machining, showing an in-process computing time typically below 1 s per image to a maximum of 2 s at the last stages of the computed industrial scenes, and a relative precision of 1/10,000 with an error RMS below 0.2 pixel at image plane, ranging at the same precision performance reported for portable photogrammetry with precise off-process pre-calibrated cameras. Efficient camera model in-process self-calibration is also demonstrated, with post-processing times ranging 11 s.

Alternatives for increasing computational in-process efficiency have been pointed out, especially focusing on large scale industrial scenarios where a high number of images and optical targets to compute can be expected. Regarding precision, anisotropy has been observed in the spatial uncertainty distribution of the optical target coordinates, pointing to a better potential for the developed system than the figures reported according to the worst-case scenario. Further steps towards the prediction of ray-net-conditioning-induced uncertainty contributions, such as analytical approaches using error propagation theory, may enable to take the most of that potential, still remaining as a relevant and challenging issue in the state-of-the-art. Additionally, further steps could be conducted for enabling precise measurements with low cost but thermo-mechanically unstable consumer grade digital cameras, where the influence of an image dependent focal length due to integrated autofocus optics might be considered for precise self-calibration.

Finally, in the actual context, the need for “smart” systems integrating software procedures and hardware systems for data acquisition, self-diagnostics, set-up, control of tolerances, etc., is becoming more and more pressing. In addition, rapid advances in computing processing, image measurement and characterization algorithms have allowed photogrammetric systems to integrate with CAD/CAM systems offering high accuracy capabilities, and more and more “real-time” (1/25 s and faster) image sequence acquisition. The photogrammetric system presented here is a step towards meeting these requirements, accomplishing accurate measurements with inexpensive, easy-to-use measuring systems and with optimized procedures that make the whole measuring process much less time consuming.

## Figures and Tables

**Table 1:** Input data for extrinsic computing: 3D coordinates (*x*, *y*, and *z* in mm, for *X*_1_ to *X*_6_) for coded targets on reference frame, and image coordinates (*h_ij_* and *v_ij_* in pixels, for image *j* = 1 to 3).

	*Reference Frame Targets* (*X_i_*)			*Image Coordinates* ($h_{ij}$,$v_{ij}$)					
	*x*	*y*	*z*	*h_i_*_1_	*v_i_*_1_	*h_i_*_2_	*v_i_*_2_	*h_i_*_3_	*v_i_*_3_
*X*_1_	0	0	0	−51.652	21.593	−21.713	−24.392	24.592	37.326
*X*_2_	−169.963	2.650	−0.356	−696.361	27.686	−10.666	−574.886	18.178	637.234
*X*_3_	170.036	0	0	594.253	7.982	−26.418	528.265	23.846	−561.086
*X*_4_	−1.742	−169.186	0	−52.039	541.249	−448.374	−41.592	504.455	37.142
*X*_5_	−0.162	26.998	145.558	−64.312	−473.212	403.334	−25.024	−431.115	32.518
*X*_6_	0.109	26.590	28.314	−54.508	−125.784	104.393	−22.956	−112.206	36.936

**Table 2:** Computed results for independent camera extrinsic approaches ($d_{x}$, $d_{y}$, and $d_{z}$ in mm, and α, β, and γ in radians), given by the input data in Table 1, along with the optimization quality index for each minimized reprojection error vector (RMS in pixels).

	*d_X_*	*d_Y_*	*d_Z_*	*α*	*β*	*γ*	*RMS*
*Image 1*	−13.552	5.620	1145.020	−2.375	0.005	0.020	0.482
*Image 2*	−6.593	−7.545	1340.136	−2.348	−0.009	−1.580	0.454
*Image 3*	6.894	10.494	1233.812	−2.378	0.023	−4.712	0.471

**Table 3:** Computed results for independent target coordinate initial approaches, given by the input data in Table 2, along with the optimization quality index for each minimized reprojection error vector (RMS in pixels).

*Target Coordinates (X_i_)*				*Image Coordinates ($h_{ij}$,$v_{ij}$)*						
*id*	*x*	*y*	*z*	*h_i_*_1_	*v_i_*_1_	*h_i_*_2_	*v_i_*_2_	*h_i_*_3_	*v_i_*_3_	*RMS*
*11*	−171.283	−118.827	−4.814	−745.019	400.151	−316.781	−623.685	362.860	679.588	0.334
*81*	123.935	−80.850	−0.931	446.849	247.991	−221.605	392.106	243.357	−423.092	0.085
*150*	−389.999	271.547	62.835	−1370.495	−757.428	673.595	−1141.890	−734.434	1274.263	0.691
*194*	254.725	−340.006	−97.490	1086.097	1369.686	−1175.381	899.456	1284.152	−994.748	1.617
*942*	48.080	393.260	65.336	84.859	−1027.708	869.982	127.453	−958.663	−95.253	1.144
*943*	37.041	366.774	50.754	52.101	−940.542	795.293	96.428	−877.274	−63.611	0.970
*944*	73.083	376.546	50.491	165.633	−959.464	808.956	195.930	−892.928	−170.040	1.008
*947*	17.904	395.752	53.643	−10.528	−998.670	847.403	43.948	−934.252	−4.878	1.039
*948*	2.228	387.889	20.125	−57.610	−895.363	759.110	0.405	−839.240	42.036	0.878

**Table 4:** Camera extrinsic parameters ($d_{x}$, $d_{y}$, and $d_{z}$ in mm, and α, β, and γ in radians) after joint bundle of epipolar net scene at Figure 9b.

	*d_X_*	*d_Y_*	*d_Z_*	α	β	γ
*Image 1*	−13.561	5.492	1145.880	−2.378	0.005	0.020
*Image 2*	−6.635	−7.598	1339.838	−2.347	−0.008	−1.580
*Image 3*	7.005	10.447	1232.964	−2.389	0.023	−4.712

**Table 5:** Coded (id) target 3D coordinates (mm) after joint bundle of scene at Figure 9b.

*id*	*x*	*y*	*z*
*11*	−170.704	−121.223	−1.921
*81*	123.995	−80.446	−1.397
*150*	−390.866	274.147	60.943
*194*	254.332	−340.750	−96.037
*942*	49.131	422.060	46.501
*943*	37.779	392.934	32.957
*944*	74.603	403.023	32.620
*947*	18.272	425.879	33.690
*948*	2.222	417.722	−0.569

**Table 6:** Camera intrinsic parameters after self-calibration.

*f* (mm)	$cl_{0}$ (pixel)	$rw_{0}$ (pixel)	$k_{1}$ (pixel^−2^)	$k_{2}$ (pixel^−4^)	$\pi_{1}$ (pixel^−1^)	$\pi_{2}$ (pixel^−1^)
24.557	−7.652	31.396	4.866 × 10^−09^	−2.150 × 10^−16^	−8.636 × 10^−08^	−1.236 × 10^−08^
